# Diagnostic accuracy of tests to detect Hepatitis C antibody: a meta-analysis and review of the literature

**DOI:** 10.1186/s12879-017-2773-2

**Published:** 2017-11-01

**Authors:** Weiming Tang, Wen Chen, Ali Amini, Debi Boeras, Jane Falconer, Helen Kelly, Rosanna Peeling, Olivia Varsaneux, Joseph D. Tucker, Philippa Easterbrook

**Affiliations:** 1 University of North Carolina Project-China, No. 2 Lujing Road, Guangzhou, 510095 China; 2 Guangdong Provincial Dermatology Hospital (Dermatology Hospital, Southern Medical University), Guangzhou, China; 3 SESH Global, Guangzhou, China; 4 School of Medicine, University of North Carolina at Chapel Hill, Chapel Hill, NC USA; 5 School of Public Health, Sun Yat-sen University, Guangzhou, China; 6 Center for Migrant Health Policy, Sun Yat-sen University, Guangzhou, China; 7 London School of Hygiene and Tropical Medicine, Keppel St, London, UK; 8 Global Hepatitis Programme, HIV Department, World Health Organization, Geneva, Switzerland

**Keywords:** Diagnostic accuracy, Diagnostic tests, Hepatitis C, HCV antibody, Rapid diagnostic tests

## Abstract

**Background:**

Although direct-acting antivirals can achieve sustained virological response rates greater than 90% in Hepatitis C Virus (HCV) infected persons, at present the majority of HCV-infected individuals remain undiagnosed and therefore untreated. While there are a wide range of HCV serological tests available, there is a lack of formal assessment of their diagnostic performance. We undertook a systematic review and meta-analysis to evaluate he diagnostic accuracy of available rapid diagnostic tests (RDT) and laboratory based EIA assays in detecting antibodies to HCV.

**Methods:**

We used the PRISMA checklist and Cochrane guidance to develop our search protocol. The search strategy was registered in PROSPERO (CRD42015023567). The search focused on hepatitis C, diagnostic tests, and diagnostic accuracy within eight databases (MEDLINE, EMBASE, the Cochrane Central Register of Controlled Trials, Science Citation Index Expanded, Conference Proceedings Citation Index-Science, SCOPUS, Literatura Latino-Americana e do Caribe em Ciências da Saúde and WHO Global Index Medicus. Studies were included if they evaluated an assay to determine the sensitivity and specificity of HCV antibody (HCV Ab) in humans. Two reviewers independently extracted data and performed a quality assessment of the studies using the QUADAS tool. We pooled test estimates using the DerSimonian-Laird method, by using the software R and RevMan. 5.3.

**Results:**

A total of 52 studies were identified that included 52,673 unique test measurements. Based on five studies, the pooled sensitivity and specificity of HCV Ab rapid diagnostic tests (RDTs) were 98% (95% CI 98-100%) and 100% (95% CI 100-100%) compared to an enzyme immunoassay (EIA) reference standard. High HCV Ab RDTs sensitivity and specificity were observed across screening populations (general population, high risk populations, and hospital patients) using different reference standards (EIA, nucleic acid testing, immunoblot). There were insufficient studies to undertake subanalyses based on HIV co-infection. Oral HCV Ab RDTs also had excellent sensitivity and specificity compared to blood reference tests, respectively at 94% (95% CI 93-96%) and 100% (95% CI 100-100%). Among studies that assessed individual oral RDTs, the eight studies revealed that OraQuick ADVANCE® had a slightly higher sensitivity (98%, 95% CI 97-98%) compared to the other oral brands (pooled sensitivity: 88%, 95% CI 84-92%).

**Conclusions:**

RDTs, including oral tests, have excellent sensitivity and specificity compared to laboratory-based methods for HCV antibody detection across a wide range of settings. Oral HCV Ab RDTs had good sensitivity and specificity compared to blood reference standards.

**Electronic supplementary material:**

The online version of this article (10.1186/s12879-017-2773-2) contains supplementary material, which is available to authorized users.

## Background

Hepatitis C is a liver disease caused by the hepatitis C virus (HCV) that causes acute and chronic infection [1, 2]. An estimated 71 million people had chronic hepatitis C infection worldwide in 2015 [3]. Viral hepatitis caused 1.34 million deaths in 2015, a number comparable to deaths caused by tuberculosis and higher than those caused by HIV [3]. The introduction of direct-acting antivirals (DAAs) has led to a sustained virological response (SVR) in greater than 90% of treated individuals [4, 5]. DAAs are now recommended by the World Health Organization (WHO) [1] and many other HCV treatment guidelines [1]. DAAs will not only improve SVR rates but also may simplify HCV management algorithms and allow smaller health facilities to manage HCV-infected individuals [6]. Despite the availability of effective treatment, most HCV-infected individuals remain undiagnosed and untreated [7]. Left untreated, approximately 15–30% of individuals with chronic HCV infection progress to cirrhosis, leading to end-stage liver disease and hepatocellular carcinoma [1, 2].

In February 2016 the WHO updated the guidelines for the screening, care, and treatment of persons with chronic hepatitis C infection [1]. These guidelines included recommendations on whom to screen for HCV and how to confirm HCV infection, but not which tests are optimal for initial screening. Advances in HCV detection technology create new opportunities for enhancing screening, referral, and treatment. Previous systematic reviews on HCV infection have focused on treatment response [8, 9], clinical complications [10], and epidemiology [11, 12]. Two previous systematic reviews on hepatitis C testing have focused on evaluating point-of-care tests compared to EIAs and other reference tests [13, 14]. We have undertaken a further systematic review and meta-analysis to generate pooled sensitivity and specificity of rapid diagnostic tests used to detect HCV antibody (HCV Ab), and to inform the development of recommendations on serological testing in the 2017 WHO testing guidelines [15].

## Methods

### Research question

The main purpose of the review was to assess the diagnostic accuracy of available assays for detecting HCV Ab in persons identified for hepatitis C testing. The research question was structured in a PICO format (ie. population, intervention, comparisons and outcome).

P: Persons identified for HCV testing; I: Rapid diagnostic tests and enzyme immunoassays for HCV Ab detection; C: 1), EIA (with a subanalysis based on the last 10 years); 2), NAT (nucleic acid testing); 3), Immunoblot or similar assay; 4), A combination of 1,2,3 above; O: Diagnostic accuracy [Sensitivity (SE), Specificity (SP), Positive predictive value (PPV), Negative predictive value (NPV), True Negative, True Positive (TP), False negative (FN), and False positive (FP)].

### Search strategy and identification of studies

Search strategies were developed by a medical librarian with expertise in designing systematic review searches. Our search algorithm consisted of the following components: hepatitis C, diagnostic tests, and diagnostic accuracy. We searched MEDLINE (OVID interface, 1946 onwards), EMBASE (OVID interface, 1947 onwards), the Cochrane Central Register of Controlled Trials (Wiley interface, current issue), Science Citation Index Expanded (Web of Science interface, 1970 onwards), Conference Proceedings Citation Index-Science (Web of Science interface, 1990 onwards), SCOPUS (1960 onwards), Literatura Latino-Americana e do Caribe em Ciências da Saúde (LILACS) (BIREME interface) and WHO Global Index Medicus. The search was supplemented by searching for ongoing studies in WHO’s International Clinical Trials Registry. The literature search was limited to English language and human subjects that available until April 30th, 2015. In addition to searching databases, we contacted individual researchers and authors of major trials to address whether any relevant manuscripts are in preparation or in press. The references of published articles found in the above databases were searched for additional pertinent materials.

Study selection proceeded in three stages: 1) titles/abstracts were screened by a single reviewer according to standard inclusion and exclusion criteria; 2) full manuscripts were obtained and evaluated by two independent reviewers to include or not; 3) two independent reviewers extracted all data. Differences were resolved by a third independent reviewer.

### Selection criteria

The inclusion criteria included the following: primary purpose was HCV Ab test evaluation, reported sensitivity and specificity of HCV Ab test kits, and studies published before May 2015. We included observational and randomised control trial (RCT) studies that provided original data from patient specimens. Studies that only reported sensitivity or specificity, conference abstracts, comments or review papers, panel studies, or those that only used reference assays for positive samples were excluded. In this manuscript, a hepatitis panel refers to a laboratory series test in which use the blood with confirmed hepatitis C serostatus to assess the accuracy of a testing kit.

### Data extraction

Information on the following variables were extracted from each individual study: first author, total sample size, country (and city) of sampling, sample type (oral fluid, finger prick, venous blood), point-of-care (POC, defined as being able to give a result within 60 min and having the results to guide clinical management in the same encounter), eligibility criteria, reference standard, manufacturer, raw cell numbers (true positives, false negatives, false positives, true negatives), antibody-antigen combo (yes or no), sources of funding, reported conflict of interest, and study population (general population, high risk population and hospitalized population). The high risk population groups include men who have sex with men, sex workers and their clients, transgender people, people who inject drugs and prisoners and other incarcerated people [16]. The hospitalized population was defined as those admitted to a hospital for medical care or observation. We also verified whether assays evaluated in the studies were currently on the market (as of June 1st, 2017), and if this was the case, we also reported the available version of the testing kit (Table 1).

**Table 1:** Characteristics of studies focused on evaluating diagnostic accuracy of HCV antibody tests

First author	Year	Settings	Sample type	Manufacturer	Study type	Sample size	POC (Y/N)	Reference standard	Still on the market? PRODUCT NAME
Al-Tahish et al.	2013	Egypt	Venous blood	HCV one step test device (ACON Laboratories, USA), Fourth- generation HCV TRI_DOT (J. Mitra Co, India) and ImmunoComb II HCV (Inverness Medical Innovations, USA)	CS	100	Y	PCR	Yes Foresight® HCV EIA test kit; Yes, HCV TRI_DOT
Bonacini et al.	2001	USA	Venous blood	Ortho Clinical Diagnostics (Raritan, NJ, USA)	CS	222	N	Chiron IMMUNOBLOT HCV 3.0 SIA	Not available
Buti et al.	2000	Sprain	Serum	Not available	CS	188	Y	IMMUNOBLOT	Not avaliable
Caudai et al.	1998	USA	Serum or plasma samples	ELISA 2nd generation Abbott Laboratories, Abbott park, IL, USA)	CS	682	N	PCR	Not avaliable
Cha et al.	2013	Korea	Oral fluids and serum	OraQuick (OraSure Technologies, PA USA)	CC	437	Y	PCR	Yes, The OraQuick® HCV
Croom et al.	2006	Austria	Venous blood	Monolisa anti-HCV PLUSVersion 2 EIA (Bio-Rad, France)	CS	182	N	EIA	Yes, MONOLISA™ Anti-HCV PLUS Assay Version 2
da Rosa et al.	2013	Brazil	Serum	Rapid Test Bioeasy® (Standard Diagnostics, Yongin, Korea) and Imuno-Rapido HCV® (Wama Diagnostica, Brazil).	CS	307	Y	Architect HCV, PCR	Not available for Rapid Test Bioeasy; Yes, Imuno-Rapido HCV
Daniel et al.	2005	India	Serum	TRI DOT (J. MITRA &Co. Ltd., New Delhi, India)	CS	2590	Y	EIA, IMMUNOBLOT, PCR	YES, HCV TRI_DOT
Denoyel et al.	2004	France and Germany	Serum or plasma samples	AxSYM HCV 3.0 (other information is not available)	CS	5700	N	IMMUNOBLOT	Yes, AXSYM HCV 3.0
Dokubo et al.	2014	USA	Blood	HCV Version 3.0 ELISA (Ortho®)	CS	132	N	PCR	Yes, ORTHO® HCV 3.0 Elisa
Drobnik et al.	2011	USA	Oral fluid	OraQuick (OraSure Technologies, PA USA)	CS	484	Y	EIA, IMMUNOBLOT	Yes, The OraQuick® HCV
Eroglu et al.	2000	Turkey	Plasma specimens	ELISA v3.0(Ortho®)	CS	160	N	PCR	Yes, ORTHO® HCV 3.0 Elisa
Feucht et al.	1995	Germany	Plasma specimens	Abbott HCV second-generation enzyme immunoassay (other information is not available)	CS	262	N	IMMUNOBLOT	Not avaliable
Gao et al.	2014	USA	Serum	OraQuick (OraSure Technologies, PA USA)	CS	289	Y	EIA	Yes, The OraQuick® HCV
Hess et al.	2014	USA	whole blood	DPP HIV-HCV-Syphilis Assay (Chembio Diagnostic Systems, Inc., Medford, NY)	CS	948	Y	EIA	Not avaliable
Hui et al.	2002	Hong kong, China	Whole blood	OraQuick (OraSure Technologies, PA USA)	CS	197	Y	EIA	Yes, The OraQuick® HCV
Ibrahim et al.	2015	Saudi Arabia	Oral fluid	OraQuick (OraSure Technologies, PA USA)	CC	160	Y	PCR	Yes, The OraQuick® HCV
Ivantes et al.	2010	Brazil	Whole blood	HCV Rapid Test Bioeasy (Bioeasy Diagnostica Ltda, Minas Gerais, Brazil)	CS	71	Y	CLIA	Not available
Jewett et al.	2012	USA	Oral fluids	Chembio DPP HCV test (Chembio Diagnostic Systems,USA) and Rapid HIV/HCV antibody test (Medmira Laboratories, Canada)	CS	407	Y	IMMUNOBLOT/NAT	Not available for Chembio DPP HCV test; Yes, Multiplo HBc/HIV/HCV
Kant et al.	2012	Germany	Whole blood	Toyo anti-HCV test (Turklab, Izmir, Turkey)	CS	185	Y	Architect HCV	Yes, anti-HCV TEST
Kaur et al.	2000	India	Serum	HCV Bidot (J. Mitra Co., India)	CS	2754	Y	EIA 3rd generation	YES, DIAGNOS HCV BI-DOT
Kim et al.	2013	Republic of Korea	Serum	GENEDIA® HCV Rapid LF (Green Cross medical science corp., Korea)	CC	200	Y	IMMUNOBLOT	Yes, GENEDIA HCV Rapid LF test kit
Kosack et al.	2014	Germany	Serum	The ImmunoFlow HCV test (Core Diagnostics,United Kingdom)	CS	81	Y	IMMUNOBLOT	Yes, ImmunoFlow HCV
Lakshmi et al.	2007	India	Blood	Beijing United Biomedical, Ortho Clinical Diagnostics, General Biologicals; other information is not avaliable	CS	69	N	PCR	Not avaliable
Larrat et al.	2012	France	FSB (fingerstick blood) and oral fluid	cEIA: the Monolisa® HCV-Ag-Ab-ULTRA (Bio-Rad, Marnes-la-Coquette, France)	CC	201	Y	PCR	Yes, MONOLISA™ HCV Ag-Ab ULTRA
Lee et al.	2010	USA	Oral fluid	OraQuick (OraSure Technologies, PA USA)	CS	572	Y	EIA, IMMUNOBLOT	Yes, The OraQuick® HCV
Lee et al.	2011	USA	Serum, plasma, venous blood, figerstick blood and oral fluid	Or Quick (OraSure Technologies, PA USA)	CS	2183	Y	EIA, IMMUNOBLOT, PCR	Yes, The OraQuick® HCV
Lee et al.	2011	USA	Oral fluid	OraQuick (OraSure Technologies, PA USA)	CS	2180, 2178	Y	EIA	Yes, The OraQuick® HCV
Maity et al.	2012	India	Serum	J Mitra & Co. Pvt. Ltd., SPAN Diagnostics Ltd. and Standard Diagnostics, INC, other information is not available	CC	100	Y	EIA	Not avaliable
Montebugnoil et al.	1999	Italy	whole blood	Anti-HCV Ab rapid test (1st IRP 75/537 by Thema Ricerca, WHO Geneva)	CC	100	Y	EIA, IMMUNOBLOT	Not avaliable
Mvere et al.	1996	Zimbabwe	Serum	HCV-SPOT (Genelabs Diagnostics, Singapore)	CS	206	Y	EIA 2nd generation, INNO-LIA HCV ab III	Not avaliable
Nalpas et al.	1992	France	Serum	Ortho Diagnostics, other information is not available	CS	62	N	PCR	Not avaliable
Njouom et al.	2006	Cameroon	Plasma	ImmunoComb® II HCV assay (Orgenics Ltd.,); ImmunoComb® II HCV assay (Orgenics Ltd., Not reported manufacturer located country)	CS	329	Y	EIA 3rd generation, PCR	Not avaliable
Nyirenda et al.	2008	Malawi	Serum	Monoelisa HCV Ag/Ab ultra-microplate EIA (Bio-Rad, France)	CS	202	Y	EIA	Yes, MONOLISA™ HCV Ag-Ab ULTRA
O’Connell et al.	2013	USA	Plasma, whole blood (normal) and whole blood (cold storge)	OraQuick (OraSure Technologies, PA USA); CORE (CORE Diagnostics, United Kingdom); Axiom (Axiom Diagnostics, Burstadt,Germany); FirstVue (AT First Diagnostic, Woodbury,NY, USA) and Instant View Cassette (Alfa Scientific Designs, Poway)	CC	674, 168	Y	EIA, IMMUNOBLOT, and when available viral load)	Yes, The OraQuick® HCV; Yes, Core HCV; Not avaliable for Axiom; Yes, FirstVue™ Hepatitis “C” Rapid Test; Yes, Instant-view™ Hepatitis C Virus (HCV) Serum Test
O’Flynn et al.	1997	Ireland, Germany, UK	Plasma and serum	AxSYM (Abbott Laboratories, other information is not available)	CC	5554, 1421, 643	N	ABBOTT MATRIX HCV, Chiron IMMUNOBLOT HCV 2.0 or 3.0	Yes, AXSYM HCV 3.0
Park et al.	2012	Korea	Serum	Vitros anti-HCV assay kits (Ortho-Clinical Diagnostics, Buckinghamshire, UK) and Elecsys (Roche Diagnostics GmbHMannheim, Germany)	CS	1008	N	IMMUNOBLOT HCV 3.0 and Cobas Ampliprep/Taqman HCV RNA	Not avaliable
Poovorawari et al.	1994	Thailand	Serum	HCV-SPOT assay (Genelabs Diagnostics Pty Ltd., Singapore)	CS	192	Y	EIA 2nd generation or IMMUNOBLOT	Not avaliable
Prayson et al.	1993	USA	Serum	C100-3 HCV EIA (Abbott Laboratories, other information is not available)	CS	123	N	IMMUNOBLOT 2.0	Not avaliable
Rihn et al.	2000	France	serum	MATRIX hcv2 (Abbott Laboratories, other information is not available)	CS	146	N	PCR	Not avaliable
Scalioni Lde et al.	2014	Brazil	Serum,whole blood and oral fluid	WAMA Imuno-Rápido HCV Kit (WAMA Diagnóstica, Brazil); Bioeasy HCV Rapid Test, (Bioeasy Diagnóstica Ltd., Brazil) and OraQuick (OraSure Technologies, PA USA)	CS	194 or 172	Y	PCR	Yes, Imuno-Rapido HCV; Not avaliable for Rapid Test Bioeasy; Yes, The OraQuick® HCV
Smith et al.	2011	USA	Whole blood, oral fluid	Multiplo Rapid HIV/HCV Antibody Test (MedMira, Canada); Chembio DPP HCV test (Chembio Diagnostic Systems, USA) and OraQuick (OraSure Technologies, USA)	CS	476, 385, 432, 549, 266	Y	MEIA/EIA/CLIA, IMMUNOBLOT	Yes, Multiplo HBc/HIV/HCV; Not avaliable for Chembio DPP HCV test; Yes, The OraQuick® HCV
Smith et al.	2011	USA	Oral fluid and blood	Multiplo Rapid HIV/HCV Antibody Test (MedMira, Canada); Chembio DPP HCV test (Chembio Diagnostic Systems, USA)	CS	1081	Y	Chiron IMMUNOBLOT HCV 3.0 SIA; Bayer Advia Centaur HCV Chemiluminescent immunoassay	Yes, Multiplo HBc/HIV/HCV; Not avaliable for Chembio DPP HCV test
Sommese et al.	2014	Italy	Blood	CMIA assays (Abbott Diagnostics, Wiesbaden, Germany)	CS	17,894	N	INNO-LIA (Innogenetics, Ghent, Belgium), NAT	Not avaliable
Tagny et al.	2014	Cameron	Plasma	HCV Ag/Ab combination assay (Monolisa HCV Ag-Ab Ultra, BioRad, Marnes La Coquette, France)	CS	1998	Y	EIA	Yes, MONOLISA™ HCV Ag-Ab ULTRA
Vrielink et al.	1996	Netherlands	Blood	Abbott HCV EIA 3.0 (Abbott laboratories, Murex anti-HCV VK47 (Murex Diagnostic) and Ortho HCV 3.0 elisa (Ortho Diagnostic Systems; other information is not available	CS	403, 212, 253 03 1055	N	PCR	Not available for Abbott HCV EIA 3.0; Yes, Murex anti-HCV (version 4); Yes, ORTHO® HCV 3.0 Elisa
Vrielink et al.	1995	Netherlands	Blood	Monolisa anti-HCV new antigens (Sanofi Diagnostics Pasteur), Abbott HCV EIA 3.0 (Abbott Laboratories); other information is not available	CS	403, 212, 253	N	PCR	Not avaliable
Yang et al.	2011	China	Serum	AxSYM HCV 3.0 (Abbott Laboratories), Murex Ag/Ab test (Abbott Laboratories); other information is not available	CC	101 or 100	N	HCV RNA test (COBAS AMPLICOR Hepatitis C Virus Test, version 2.0	Yes, AXSYM HCV 3.0; Not avaliable for Murex Ag/Ab test
Yang et al.	2013	China	Serum	Elecsys anti-HCV II (Roche Diagnostics GmbH), Architect anti-HCV (Abbott) and Vitros anti-HCV (Ortho-Clinical Diagnostics), other information is not available	CS	859 or 167	N	IMMUNOBLOT 3.0 test or the Realtime HCV RNA assay	Yes, Elecsys® Anti-HCV II; Yes, ARCHITECT i1000SR I; Not available for Ortho-Clinical Diagnostics
Yarri et al.	2006	Israel	Serum and oral fluid	ImmunoComb II HCV (Inverness Medical Innovations, USA)	CS	37	Y	PCR	Not avaliable
Yoo et al.	2015	South Korea; China; China/Taiwan; Thailand; Australia; Malaysia; Indonesia	Serum	Elecsys® Anti-HCV II assay; (Roche Diagnostics GmbH, other information is not avaliable)	CS	7726	Y	1 or more of the following comparator assays at 9 centers: ARCHITECTTM Anti-HCV; Serodia®-HCV Particle Agglutination; Vitros® ECi Anti-HCV; Elecsys® Anti-HCV; ADVIA Centaur® HCV; InTec® HCV EIA; or Livzon® Anti-HCV.	Yes, Elecsys® Anti-HCV II
Yuen et al.	2001	China	Serum	SM-HCV Rapid Test (SERO-Med Laborspezialita¨ten GmbH, Eichsta ¨tt, Germany)	CC	290	Y	EIA, PCR	Not avaliable

Notes: *CC* case–control study, *CS* cross-sectional study

### Assessment of methodological quality

Study quality was evaluated using the QUADAS-2 tool [17] and the STARD checklist [18]. QUADAS includes domains to evaluate bias in the following categories: risk of bias (patient selection, index test, reference standard, flow, and timing); applicability concerns (patient selection, index test, reference standard). The STARD checklist consists of a checklist of 25 items and flow diagram that authors can use to ensure that all relevant information is present.

### Data analysis and synthesis

#### Data synthesis

Data were extracted to construct 2 × 2 tables. By comparing with reference standard results, the index test results were categorized as a true positive, a false positive, a false negative, or a true negative. Indeterminate test results were not included in pooled analyses.

#### Statistical analysis

To estimate test accuracy, we calculated sensitivity and specificity for each study and pooled statistics, along with 95% confidence intervals [19]. We pooled test estimates using the DerSimonian-Laird method, a bivariate random effect model. We did further subanalyses based on reference standard (EIA alone; NAT or immunoblot; EIA, NAT, or immunoblot), brand, sample type, and combination test. We performed all statistical analysis (including heterogeneity, through Q test) using the software R and RevMan 5.3.

## Results

### Study selection

**Fig. 1:**
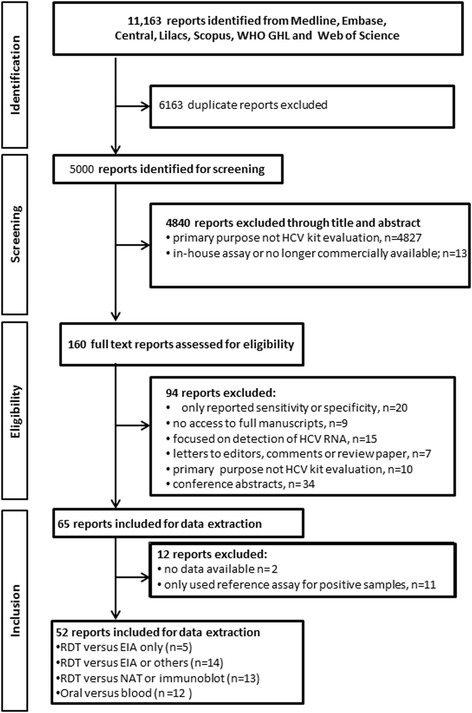
PRISMA flow diagram outlining study selection examining the diagnostic accuracy of HCV antibody tests

A total of 11,163 citations were identified, and 6163 duplicates were removed. Each of the 5000 unique citations was examined. A total of 52 research studies were included in the final analysis (Fig. 1) [8, 16, 19–68]. Of the 52 studies, 32 studies evaluated the accuracy of 30 different rapid diagnostic tests (RDTs) [19–50], of which 5 evaluated RDTs compared to EIA alone [25, 26, 31, 34, 49], 13 compared RDT results to NAT or immunoblot [19–22, 27, 29, 32, 37, 42, 43, 45, 47, 50], and 14 focused on evaluating RDT by comparing with the results of EIA or immunoblot or NAT [23–26, 30, 34, 35, 38, 39, 41, 44, 48, 49, 51]. Eleven studies evaluated the diagnostic accuracy of oral fluid RDTs [22, 24, 27, 29, 33, 34, 43–45, 47, 52].

There were insufficient data to undertake a subanalysis based on HIV co-infection or other co-infections.

### Study characteristics

Of the 52 included studies, nine were published before 2000 [37, 38, 42, 53–58], 12 studies reported evaluation using oral fluid samples, and 34 studies evaluated POC tests. Of the 52 studies, 41 different brands of testing kits were evaluated (Table 1).

### Assessment of the quality of the studies

All studies used a cross-sectional or case–control design. The risk of bias in patient selection, index test, or reference standard was assessed using QUADAS-2 (Table 2). Among the included studies, 25 had at least one category that was considered high risk [19, 22, 25–28, 30, 31, 34, 36–39, 41, 45–50, 53, 55, 56, 58–62]. The risk of bias in patient selection usually came from a poor description of patient selection and clinical scenario. Bias in the index test was primarily due to a lack of reported blinding while reading test results. Bias in the reference standard was due to the use of multiple reference standards (EIA, NAT, and/or immunoblot). Bias in the flow and timing was primarily due to a lack of reported details.

**Table 2:** Quality assessment by QUADAS-2 of the included studies

Reports		Bias assessment/Risk of bias				Acceptability concerns		
		Patient selection	Index test	Reference Standard	Flow and timing	Patient selection	Index test	Reference Standard
Al-Tahish et al.	2013	UC	LR	LR	LR	LR	LR	LR
Bonacini et al.	2001	HR	LR	LR	LR	LR	LR	LR
Buti et al.	2000	UC	UC	LR	LR	HR	LR	LR
Caudai et al.	1998	HR	LR	LR	LR	UC	LR	LR
Cha et al.	2013	HR	LR	LR	LR	UC	LR	LR
Croom et al.	2006	LR	LR	LR	UC	LR	LR	LR
da Rosa et al.	2013	HR	UC	LR	LR	HR	UC	LR
Daniel et al.	2005	LR	LR	LR	LR	LR	LR	LR
Denoyel et al.	2004	UC	LR	LR	LR	UC	LR	HR
Drobnik et al.	2011	LR	UC	LR	UC	LR	UC	LR
Eroglu et al.	2000	LR	LR	LR	LR	LR	LR	LR
Feucht et al.	1995	HR	LR	LR	LR	HR	LR	LR
Gao et al.	2014	LR	LR	LR	HR	LR	LR	LR
Hess et al.	2014	LR	HR	LR	LR	LR	HR	LR
Hui et al.	2002	HR	LR	HR	LR	HR	LR	HR
Ivantes et al.	2010	LR	UC	HR	LR	LR	LR	HR
Jewett et al.	2012	LR	LR	LR	LR	LR	LR	LR
Dokuboa et al.	2014	UC	LR	LR	LR	UC	LR	LR
Kant et al.	2012	HR	UC	HR	LR	HR	UC	HR
Kaur et al.	2000	LR	UC	HR	LR	LR	LR	LR
Kim et al.	2013	UC	LR	LR	LR	UC	LR	LR
Kosack et al.	2014	HR	LR	LR	LR	HR	LR	LR
Lakshmi et al.	2007	UC	LR	LR	UC	HR	LR	LR
Larrat et al.	2012	LR	LR	LR	LR	LR	LR	LR
Lee et al.	2010	LR	UC	LR	LR	LR	UC	LR
Lee et al.	2011	HR	UC	LR	LR	LR	LR	LR
Maity et al.	2012	HR	UC	HR	LR	HR	UC	HR
Montebugnoil et al.	1999	HR	LR	LR	LR	HR	LR	LR
Mvere et al.	1996	HR	LR	LR	LR	HR	LR	LR
Nalpas et al.	1992	HR	LR	LR	UC	HR	LR	LR
Njouom et al.	2006	HR	UC	LR	LR	HR	UC	LR
Nyirenda et al.	2008	LR	UC	LR	LR	LR	LR	LR
O’Connell et al.	2013	HR	LR	HR	LR	HR	LR	LR
O’Flynn et al.	1997	UC	LR	LR	UC	LR	LR	LR
Park et al.	2012	UC	LR	LR	UC	LR	LR	LR
Poovorawari et al.	1994	LR	UC	LR	LR	LR	LR	LR
Prayson et al.	1993	UC	LR	LR	UC	UC	LR	LR
Rihn et al.	2000	UC	LR	LR	UC	UC	LR	LR
Scalioni et al.	2014	UC	LR	LR	UC	UC	LR	LR
Smith et al.	2011	LR	LR	LR	LR	LR	LR	LR
Smith et al.	2011	HR	LR	LR	LR	HR	LR	LR
Sommese et al.	2014	LR	LR	LR	LR	LR	LR	LR
Lee et al.	2010_2	LR	LR	LR	LR	LR	LR	LR
Ibrahim et al.	2015	HR	LR	LR	LR	HR	LR	LR
Tagny et al.	2014	LR	UC	HR	LR	LR	UC	HR
Vrielink et al.	1995	UC	LR	LR	LR	UC	LR	LR
Vrielink et al.	1995_2	UC	LR	LR	LR	HR	LR	LR
Yang et al.	2011	UC	LR	LR	LR	UC	LR	LR
Yang et al.	2013	LR	LR	LR	UC	LR	LR	LR
Yarri et al.	2006	HR	LR	LR	LR	HR	LR	LR
Yoo	2015	UC	LR	LR	HR	UC	LR	LR
Yuen et al.	2001	HR	LR	LR	LR	HR	LR	LR

N;otes: *LR* low risk, *HR* high risk, *UC* unclear risk

### Diagnostic accuracy

#### Overall clinical performance of assays

**Fig. 2:**
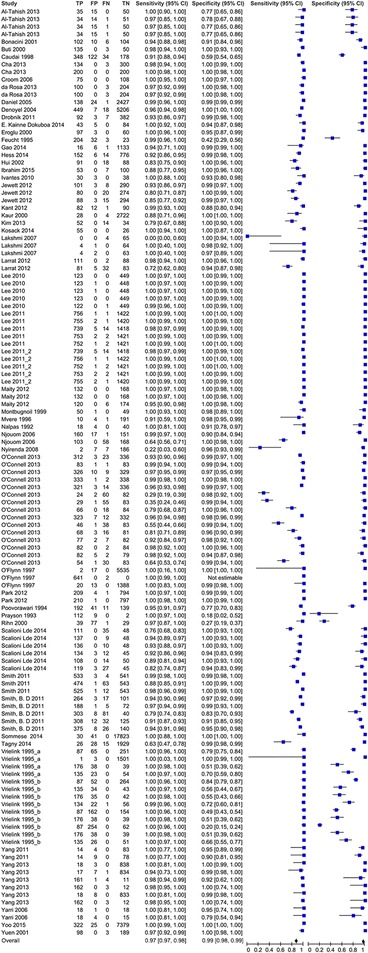
Sensitivity and specificity of HCV Ab tests included in the review (*n* = 52)

The 52 included studies contributed 127 data points from 52,273 unique test measurements. Some studies contributed additional data points by comparing the accuracy of two or more tests, reporting data from multiple study sites, or reporting the accuracy of a test in more than one type of specimen. The sample sizes of the included studies ranged from 37 to 17,894. Sensitivities of included studies ranged from 22 to 100%, and specificities ranged from 77 to 100%. The overall pooled sensitivity and specificity for all tests were 97% (95% CI: 97%–98%) and 99% (95% CI: 98%-99%) respectively. Figure 2 shows estimates of sensitivity and specificity from each study.

#### Manufacturers and accuracy of RDTs among included studies

Overall, 32 studies evaluated the accuracy of 30 different RDTs (Table 3). The most commonly evaluated test kit was the OraQuick ADVANCE® from OraSure Technologies.

**Table 3:** Manufacturers and accuracy of RDTs among included studies

First author	Manufacturer	Sample size	TP	FP	TN	FN	SE	SP
Montbugnoil et al.	Anti-HCV Ab rapid test (1st IRP 75/537 by Thema Ricerca, WHO Geneva)	100	50	1	49	0	100%	98%
O’Connell, R. J. et al.	Axiom (Axiom Diagnostics, Burstadt,Germany)	674	326	10	329	9	97%	97%
O’Connell, R. J. et al.	Axiom (Axiom Diagnostics, Burstadt,Germany)	168	77	2	82	7	92%	98%
O’Connell, R. J. et al.	Axiom (Axiom Diagnostics, Burstadt,Germany)	168	82	5	79	2	98%	94%
Scalioni Lde, P et al.	Bioeasy HCV Rapid Test, (Bioeasy Diagnóstica Ltd., Brazil)	194	137	0	48	9	94%	100%
Scalioni Lde, P et al.	Bioeasy HCV Rapid Test (Bioeasy Diagnóstica Ltd., Brazil)	194	111	0	48	35	76%	100%
Scalioni Lde, P et al.	Bioeasy HCV Rapid Test (Bioeasy Diagnóstica Ltd., Brazil)	194	136	0	48	10	93%	100%
Jewett, A et al.	Chembio DPP HCV Test (Chembio Diagnostic Systems, USA)	407	101	3	290	8	93%	99%
Jewett, A et al.	Chembio DPP HCV test (Chembio Diagnostic Systems,USA)	400	88	3	294	15	85%	99%
Smith, B. D et al.	Chembio DPP HCV test (Chembio Diagnostic Systems, USA)	476	308	12	125	32	91%	91%
Smith, B. D et al.	Chembio DPP HCV test (Chembio Diagnostic Systems, USA)	385	264	3	101	17	94%	97%
Smith et al. et al.	Chembio DPP HCV test (Chembio Diagnostic Systems, USA)	1081	525	1	543	12	98%	100%
O’Connell, R. J. et al.	CORE (CORE Diagnostics, United Kingdom)	168	29	1	83	55	35%	99%
O’Connell, R. J et al.	CORE (CORE Diagnostics, United Kingdom)	168	24	2	82	60	29%	98%
O’Connell, R. J et al.	CORE (CORE Diagnostics, United Kingdom)	674	323	7	332	12	96%	98%
Maity et al.	Diagnostics Ltd. (other information is not available)	300	132	0	168	0	100%	100%
O’Connell, R. J. et al.	FirstVue (AT First Diagnostic, Woodbury,NY, USA)	168	66	0	84	18	79%	100%
O’Connell, R. J. et al.	FirstVue (AT First Diagnostic, Woodbury,NY, USA)	168	54	1	83	30	64%	99%
O’Connell, R. J. et al.	FirstVue (AT First Diagnostic, Woodbury,NY, USA)	674	312	3	336	23	93%	99%
Al-Tahish et al.	Fourth- generation HCV TRI_DOT (J. Mitra Co, India)	100	34	15	50	1	97%	77%
Daniel et al.	Fourth- generation HCV TRI_DOT (J. Mitra Co, India)	2590	138	24	2427	1	99%	99%
Kim, M. H. et al.	GENEDIA® HCV Rapid LF (Green Cross medical science corp., Korea)	100	52	0	34	14	79%	100%
Kaur et al.	HCV Bidot (J. Mitra Co., India)	2754	28	0	2722	4	88%	100%
Al-Tahish	HCV one step test device (ACON Laboratories, USA)	100	34	15	50	1	97%	77%
Ivantes et al.	HCV Rapid Test Bioeasy (Bioeasy Diagnostica Ltd., Brazil)	71	30	3	38	0	100%	93%
da Rosa et al.	HCV Rapid Test Bioeasy® (Standard Diagnostics, South Korea)	307	100	0	204	3	97%	100%
Poovoran et al.	HCV-SPOT assay (Genelabs Diagnostics Pty Ltd., Singapore)	192	41	11	139	1	98%	93%
Mvere et al.	HCV-SPOT assay (Genelabs Diagnostics Pty Ltd., Singapore)	206	10	4	191	1	91%	98%
Njouom et al.	Hexagon® HCV (Not reported manufacturer located country)	329	160	17	151	1	99%	90%
Al-Tahish et al.	ImmunoComb II HCV (Inverness Medical Innovations, USA)	100	34	14	51	1	97%	78%
Yarri et al.	ImmunoComb II HCV (Inverness Medical Innovations, USA)	37	18	4	15	0	100%	79%
Yarri et al.	ImmunoComb II HCV (Inverness Medical Innovations, USA)	37	18	1	18	0	100%	95%
Njouom et al.	ImmunoComb® II HCV assay (Orgenics Ltd., not reported manufacturer located country)	329	103	0	168	58	64%	100%
da Rosa et al.	Imuno-Rapido HCV® (Wama Diagnostica, Brazil).	307	100	0	204	3	97%	100%
O’Connell, R. J. et al.	Instant View Cassette (Alfa Scientific Designs, Poway, CA, USA)	674	321	3	336	14	96%	99%
O’Connell, R. J. et al.	Instant View Cassette (Alfa Scientific Designs, Poway, CA, USA)	168	68	3	81	16	81%	96%
O’Connell, R. J. et al.	Instant View Cassette (Alfa Scientific Designs, Poway, CA, USA)	168	46	1	83	38	55%	99%
Maity et al.	J Mitra Co. India other information is not available)	300	120	0	174	6	95%	100%
Jewett, A et al.	Rapid HIV/HCV antibody test (Medmira Laboratories, Canada)	374	80	0	274	20	80%	100%
Nyirenda et al.	Monoelisa HCV Ag/Ab ultra-microplate EIA (Bio-Rad, France)	202	2	7	186	7	22%	96%
Tagny et al.	Monolisa HCV Ag-Ab Ultra, (BioRad, France)	1998	26	28	1929	15	63%	99%
Smith et al.	Multiplo Rapid HIV/HCV Antibody Test (MedMira, Canada)	1081	474	1	543	63	88%	100%
Smith, B. D et al.	Multiplo Rapid HIV/HCV Antibody Test (MedMira, Canada)	432	303	8	40	81	79%	83%
Cha, Y. J. et al.	OraQuick (OraSure Technologies, PA USA)	437	134	0	300	3	98%	100%
Cha, Y. J. et al.	Architect (Abbott Laboratories, Abbott Park, IL, USA)	400	200	0	200	0	100%	100%
Lee, S. R et al.	OraQuick (OraSure Technologies, PA USA)	2183	756	1	1422	1	100%	100%
Lee, S. R et al.	OraQuick (OraSure Technologies, PA USA)	2183	755	2	1420	1	100%	100%
Lee, S. R et al.	OraQuick (OraSure Technologies, PA USA)	2183	753	2	1421	2	100%	100%
Lee, S. R et al.	OraQuick (OraSure Technologies, PA USA)	2183	752	1	1421	2	100%	100%
Lee, S. R et al.	OraQuick (OraSure Technologies, PA USA)	2183	739	5	1418	14	98%	100%
O’Connell, R. J et al.	OraQuick (OraSure Technologies, PA USA)	674	333	1	338	2	99%	100%
O’Connell, R. J et al.	OraQuick (OraSure Technologies, PA USA)	168	83	1	83	1	99%	99%
O’Connell, R. J et al.	OraQuick (OraSure Technologies, PA USA)	168	82	0	84	2	98%	100%
Smith, B. D et al.	OraQuick (OraSure Technologies, PA USA)	549	375	8	140	26	94%	.95%
Smith, B. D et al.	OraQuick (OraSure Technologies, PA USA)	266	188	1	72	5	97%	99%
Lee et al.	OraQuick (OraSure Technologies, PA USA)	572	122	0	449	1	99%	100%
Lee et al.	OraQuick (OraSure Technologies, PA USA)	572	123	0	449	0	100%	100%
Lee et al.	OraQuick (OraSure Technologies, PA USA)	572	123	0	449	0	100%	100%
Lee et al.	OraQuick (OraSure Technologies, PA USA)	572	123	1	448	0	100%	100%
Lee et al.	OraQuick (OraSure Technologies, PA USA)	572	123	1	448	0	100%	100%
Smith et al.	OraQuick (OraSure Technologies, PA USA)	1081	533	3	541	4	99%	99%
Drobnik et al.	OraQuick (OraSure Technologies, PA USA)	484	92	3	382	7	93%	99%
Stephen R. Lee et al.	OraQuick (OraSure Technologies, PA USA)	2180	756	1	1422	1	100%	100%
Stephen R. Lee et al.	OraQuick (OraSure Technologies, PA USA)	2178	755	2	1420	1	100%	100%
Stephen R. Lee et al.	OraQuick (OraSure Technologies, PA USA)	2178	753	2	1421	2	100%	100%
Stephen R. Lee et al.	OraQuick (OraSure Technologies, PA USA)	2176	752	1	1421	2	100%	100%
Stephen R. Lee et al.	OraQuick (OraSure Technologies, PA USA)	2176	739	5	1418	14	98%	100%
Gao et al.	OraQuick (OraSure Technologies, PA USA)	1156	16	6	1133	1	94%	99%
Ibrahim	OraQuick (OraSure Technologies, PA USA)	160	53	0	100	7	88%	100%
Scalioni Lde, P_2014	OraQuick (OraSure Technologies, PA USA)	172	108	0	50	14	89%	100%
Hess et al.	DPP HIV-HCV-Syphilis Assay (Chembio Diagnostic Systems, Inc., Medford, NY).	948	152	6	776	14	92%	99%
Buti et al.	Not available	188	135	0	50	3	98%	100%
Yuen et al.	SM-HCV Rapid Test (SERO-Med Laborspezialita¨ten GmbH, Eichsta ¨tt, Germany)	290	98	0	189	3	97%	100%
Maity et al.	SPAN Diagnostics, Indi, other information is not available	300	132	0	168	0	100%	100%
Kant et al.	Toyo anti-HCV test (Turklab, Izmir, Turkey)	185	82	12	90	1	99%	88%
Kosack, C. S. et al.	The ImmunoFlow HCV test (Core Diagnostics,United Kingdom)	82	55	0	26	0	100%	100%
Scalioni Lde et al.	WAMA Imuno-Rápido HCV Kit (WAMA Diagnóstica, Brazil)	194	119	3	45	27	82%	94%
Scalioni Lde, P et al.	WAMA Imuno-Rápido HCV Kit (WAMA Diagnóstica, Brazil)	194	134	3	45	12	92%	94%
Hui et al.	Not reported	197	91	0	88	18	83%	100%

#### Pooled test accuracy for RDT versus EIA alone

Overall, five studies evaluated RDTs compared to the EIA alone, with a total sample of 15,943. Of the five studies, sample sizes ranged from 197 to 2754, sensitivities ranged from 83 to 100%, and specificities ranged from 99 to 100%. The pooled sensitivity and specificity were 98% (95% CI 98%-100%) and 100% (95% CI 100%-100%), respectively, while heterogeneity was observed in the included studies (*P* < 0.001) (Table 3, Additional file 1).

For the three studies that were conducted within the last 10 years [25, 49, 51], the total sample size was 12,992, with pooled sensitivity and specificity of 99% (95%CI 99%-100%) and 100% (95%CI 100%-100%), respectively.

#### RDT accuracy compared to NAT or immunoblot

Overall, 13 studies evaluated RDTs compared to NAT or immunoblot [19–22, 27, 29, 32, 37, 42, 43, 45, 47, 50], with a total sample of 7083. Among these studies, sample sizes ranged from 36 to 549, sensitivities ranged from 76 to 100%, and specificities ranged from 77% to 100%. The pooled sensitivity and specificity were 93% (95% CI 91%-95%) and 98% (95% CI 98%- 99%), respectively, while heterogeneity was observed in the included studies (*P* < 0.001) (Table 3, Additional file 2).

#### RDT test accuracy compared to EIA, NAT or Immunoblot

Overall, 14 studies evaluated RDTs by referencing to EIA with NAT and/or immunoblot [25, 26, 31, 33–35, 38, 39, 41, 45, 48, 49], with a total sample of 42,212. Of the 14 studies, sample sizes ranged from 168 to 2754, sensitivities ranged from 29 to 100%, and specificities ranged from 90 to 100%. The pooled sensitivity and specificity were 97% (95% CI 96% -98%) and 100% (95% CI 100%-100%), respectively, while heterogeneity was observed in the included studies (*P* < 0.001) (Table 3, Additional file 3).

### Pooled test accuracy for oral versus blood samples

#### EIAs using oral fluid samples

Overall, 11 studies compared the accuracy of EIAs using oral fluid samples to a blood sample as a reference, with a total sample size of 12,370 [22, 24, 27, 29, 33, 34, 43–45, 47, 52]. Of the 12 studies, sample sizes ranged from 37 to 2176, sensitivities ranged from 72 to 100%, and specificities ranged from 91 to 100%. The pooled sensitivity and specificity were 94% (95% CI 93%-96%) and 100% (95% CI 99%-100%), respectively. Heterogeneity was observed in the included studies (*P* < 0.001) (Table 3, Additional file 4).

#### Blood samples

**Fig. 3:**
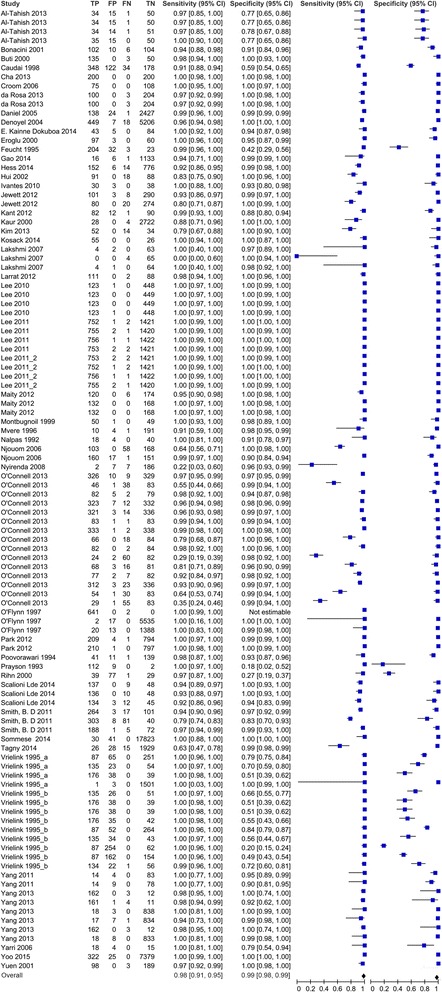
Pooled HCV Ab test accuracy for blood samples (*n* = 47 studies)

Overall, 47 studies used blood samples for evaluations, with a total sample of 90,008. Sample sizes ranged from 37 to 17,894, sensitivities ranged from 29 to 100%, and specificities ranged from 18 to 100%. The pooled sensitivity and specificity were 98% (95% CI 97%-98%) and 98% (95% CI 98%- 98%), respectively. Heterogeneity was observed in the included studies (*P* < 0.001) (Table 3, Fig. 3).

### Pooled test accuracy for OraQuick versus other brands on oral kits

#### OraQuick

Overall, eight studies reported sensitivity and specificity of OraQuick (OraSure Technologies, PA, USA), with a total sample of 9024 [22, 24, 27, 33–35, 43, 45]. The sample size of these studies ranged from 172 to 2183, sensitivities ranged from 90% to 100%, and specificities ranged from 95% to 100%. The pooled sensitivity and specificity were 98% (95% CI 97%-99%) and 100% (95% CI 90%-100%), respectively. Heterogeneity was observed in the included studies (*P* < 0.001) (Table 3, Additional file 5).

Overall, six studies reported sensitivity and specificity for other three brands of oral kits [29, 43–45, 47, 52], with a total sample of 6652. The sample size of these studies ranged from 37 to 1081, sensitivities ranged from 72 to 100%, and specificities ranged from 91 to 100%. The pooled sensitivity and specificity were 88% (95% CI 84%-92%) and 99% (95% CI 99%- 100%), respectively, while heterogeneity was observed between the included studies (*P* < 0.001) (Table 3, Additional file 6).

### Other findings

Our study further found that the overall sensitivity and specificity of studies conducted among general populations were 95% (95% CI 94%-96%) and 99% (95% CI 98%-99%), among high risk populations were 97% (95% CI 96%-98%) and 94% (95% CI 94%-95%), and among hospital patients were 97% (95% CI 96%-98%) and 100% (95% CI 100%-100%), respectively. The overall sensitivity and specificity of the antibody and antigen combo test were 86% (95% CI 79%-99%) and 99% (95% CI: 98%-100%).

### GRADE approach (Grading of Recommendations, Assessment, Development and Evaluation to assessing overall quality of evidence

#### GRADE for RDT versus EIA

HCV Ab RDTs showed comparable sensitivity and specificity compared to that of EIAs. Among the five studies that evaluated RDTs versus EIA, 15,943 of samples were evaluated, and moderate risk of bias was observed (Table 4), but there was a consistent high level of specificity. Since the unit of the analysis varied among studies (Table 4), indirectness was observed. In addition, the overall strength of the pooled evaluation was moderate, with pooled sensitivity and specificity of 99% (95% CI 98%-100%) and 100% (95% CI 100%-100%), respectively. Under the pre-test probability of 5%, the post-test probability after a positive test result is 97%, and the post-test probability after a negative test result is 100%.

**Table 4:** Pooled test accuracy for different testing strategies (*n* = 52 studies)*

Comparison	Pooled SE	95%CI		Tau-square *P*-value for hetero-geneity	Pooled SP	95%CI		Tau-square *P*-value for hetero-geneity
RDT versus EIA only (*n =* 5)	99%	98%	100%	<0.001	100%	100%	100%	<0.001
RDT versus NAT or Immunoblot (*n* = 13)	93%	91%	95%	<0.001	98%	97%	99%	<0.001
RDT versus EIA, NAT or Immunoblot (*n* = 14)	97%	96%	098%	<0.001	100%	100%	100%	<0.001
Oral RDT versus blood reference (*n* = 12)	94%	93%	96%	<0.001	100%	100%	100%	<0.001
Sample type								
Blood samples (*n* = 45)	98%	97%	98%	<0.001	98%	98%	99%	
Oral samples (*n* = 12)	94%	93%	96%	<0.001	100%	100%	100%	<0.001
Source population								
General screening (*n* = 17)	95%	94%	96%	<0.001	99%	98%	99%	<0.001
High risk population (*n* = 19)	97%	96%	98%	<0.001	94%	94%	95%	<0.001
Hospital patients (*n* = 16)	97%	96%	98%	<0.001	100%	100%	100%	<0.001
Antibody and Antigen Combo testing (*n* = 6)	86%	79%	94%	<0.001	99%	98%	100%	<0.001
Oral kits brand							100%	
OraQuick (*n* = 8)	98%	97%	99%	<0.001	100%	100%	100%	<0.001
Other brands (*n* = 6)	88%	84%	92%	<0.001	99%	99%	100%	<0.001

Notes: *Studies conducted in both LMIC and high-income countries were not included here

Studies conducted cross these regions were not included here

*SE* sensitivity, *SP* specificity

#### GRADE for oral RDT versus blood reference

The use of oral RDTs HCV Ab had comparable sensitivity and specificity compared to blood reference standards (Additional file 7). For the 12 studies evaluated oral RDT versus blood reference, 14,547 samples were evaluated. A moderate risk of bias was observed. Inconsistency was present for sensitivity, as the sensitivities of the included studies varied. But there was a consistent high level of specificity. Since the unit of the analysis varied with each other among the included studies (Table 4), indirectness was observed for included studies. In addition, the overall strength of the pooled evaluation was moderate, with pooled sensitivity and specificity of 94% (95% CI 93%-96%) and 100% (95% CI 100%-100%), respectively. Assuming a pre-test probability of 5%, the post-test probability after a positive test result was 94%, and the post-test probability after a negative test result was 100%.

## Discussion

There is a global need to expand HCV diagnostic testing. In this meta-analysis, we found HCV Ab RDTs, including those using oral fluid, showed a high overall sensitivity and specificity compared to laboratory-based EIAs. This extends the literature by including several new studies that were not included in prior reviews, including a sub-analysis that focused on use of RDTs with oral fluid. In addition, the evidence collected from this review was used to inform recommendations in the 2017 WHO guidelines on testing for hepatitis B and C [15]. The evidence for generally high levels of diagnostic accuracy across most brands from this systematic review and meta-analysis supported a strong recommendation for the use of HCV RDTs in WHO testing guidelines [15].

Our data suggest that RDTs can be used for HCV Ab detection in a wide range of clinical settings. For example, for all the included studies, 17 were conducted among general populations, 20 were among high risk populations, and 17 were among hospitalized patients (two studies included two kinds of populations). High HCV Ab RDTs sensitivity and specificity were observed across multiple different populations (including general population, high risk populations, and hospital patients), which is consistent with previous systematic reviews [13, 14, 63]. The use of an EIA to detect HCV Ab followed by NAT to confirm active infection is standard practice for diagnosis of HCV infection and recommended by the US Centers for Disease Control and Prevention and the WHO [64, 65]. However, despite these recommendations, HCV Ab EIA assays have not been widely used because of the complexity of laboratory-based assays, long turnaround time, high cost and requirements for specialized apparatus and trained technicians [13]. To overcome these barriers, RDTs for HCV Ab screening were developed [66]. They obviate the need for multiple follow-up appointments, shorten wait times, and allow for the simplification and decentralization of testing (Additional file 8). However, it is essential for policymakers, government officials, and health care practitioners engaged in HCV screening, care, and treatment to be aware that the performance of individual RDTs for detection of HCV Ab vary widely. Individual diagnostic accuracy for specific brands should be examined to ensure acceptable performance.

Our data suggest that oral fluid RDTs have high sensitivity and specificity. This is consistent with other literature [67]. Tests that can be used with non-invasive samples allow testing to be decentralized further and can be used in outreach settings [68]. Our data suggest that oral tests have a slightly lower pooled sensitivity (94%, 95%CI: 93%-96%) compared to blood-based tests (98%, 95% CI: 97%-98%) but comparable specificity. Oral HCV Ab RDTs tests may be particularly useful in contexts where venepuncture may be difficult, such as subsets of people who inject drugs which have difficult veins to access.

With the increasing availability of DAAs, countries are seeking testing kits with high sensitivity and specificity, in order to allow them to scale up HCV Ab screening, especially among at-risk populations. The advantages and disadvantages of EIAs and RDTs are well established [15]. Performance, cost, and accessibility need to be considered. Determining which tests to deploy at which level of the health care system and for what settings require policy makers to consider the different attributes of laboratory-based EIA versus blood-based or oral RDTs. Potential trade-offs include slightly lower accuracy for greater uptake and acceptability of testing, provision of test results, and linkage to care. Each country needs to decide on which trade-offs or compromises are acceptable, based not only on disease prevalence and the health care infrastructure but also on technical, socioeconomic, cultural, behavioral considerations. For example, they need to be clear on whether it is acceptable to buy Test X which is 10% less accurate than Test Y but is considerably cheaper so that many more people can be tested. In addition, although oral RDTs are less accurate than blood-based RDTs, it may be that oral RDTs will be more acceptable for outreach testing and accessing at-risk populations and allow the control programs to identify more HCV cases. In a low prevalence setting, even a test with 98% specificity can yield more false positive than true positive results. All these trade-offs can be modeled to give an estimate of the cost-effectiveness and potential impact of different strategies for HCV Ab screening.

Our review also underlines some of the common methodological problems encountered in evaluating diagnostic accuracy. Cross-sectional or case–control designs were used by all 52 included studies, introducing a potential risk of bias. These studies used a broad range of reference standards, which makes the pooled performance data less meaningful. Within the evaluation of diagnostic accuracy, even cross-sectional studies in patients with diagnostic uncertainty and direct comparison of test results with an appropriate reference standard can be considered high quality [69]. The majority of the included studies used convenience sampling. In this review, we excluded panel studies because they are not based on clinical settings and our purpose was to generate data that would be relevant in clinical settings as part of detection of HCV Ab.

Most studies that reported HIV or HBV co-infection only reported the test performance of the kits among all samples, instead of disaggregated diagnostic accuracy. There were insufficient data from two studies to undertake a subanalysis based on HIV co-infection. It may be important for policymakers to know the diagnostic accuracy of HCV Ab tests among individuals with co-infections, particularly HIV co-infection [70], and this requires further research among co-infected individuals.

Our study is subject to several limitations. First, we included studies conducted among the general population, hospital patients, and high risk populations. Diagnostic performance can be influenced by disease prevalence and HCV prevalence is variable among these different populations [71, 72]. Second, we detected substantial heterogeneity that could influence our confidence in the review findings [73], but addressed this problem through a series of sub-group stratified analyses. Third, about 20 brands of RDT kits were used in the included studies, and their performance varies considerably. This limited our ability to summarize the accuracy of different brands, with the exception of comparing OraQuick to other brands. Another concern is publication bias, as studies with poor test performance may be less likely to be published, leading to distorted estimates of accuracy [74]. Fourth, since not all HCV RDTs can be performed from oral fluid/capillary whole blood (some require plasma/serum), and some of them require a cold chain for storage and transport, the direct comparison between EIA and RDTs in this meta-analysis would be less meaningful. Fifth, we should note that not all test kits are still on the market and that versions of the tests included in this meta-analysis may have since changed. Finally, statistical heterogeneity was present. But is common in meta-analyses of diagnostic studies. Additional research is important for understanding why the tests perform more poorly in certain populations or settings.

## Conclusion

RDTs, including oral tests, have excellent sensitivity and specificity compared to laboratory-based methods for HCV antibody detection across a wide range of settings. National policymakers should consider the performance, cost and accessibility of RDTs into consideration, when selecting assays for use in their national testing algorithms.

## Additional files

Additional file 1: Pooled test accuracy of HCV Ab RDTs compared to an EIA reference (5 studies). (DOCX 360 kb)

Additional file 2: Pooled test accuracy of HCV Ab RDTs compared to a NAT or immunoblot reference (*n* = 13 studies). (DOCX 621 kb)

Additional file 3: Pooled test accuracy of HCV Ab RDTs compared to EIA, NAT or immunoblot reference standards (*n* = 14 studies). (DOCX 1170 kb)

Additional file 4: Pooled test accuracy for oral HCV Ab RDTs compared to blood as a reference (*n* = 11 studies). (DOCX 349 kb)

Additional file 5: Pooled test accuracy for HCV Ab OraQuick kits (*n* = 8 studies). (DOCX 414 kb)

Additional file 6: Pooled test accuracy for other brands of oral HCV Ab test kits (*n* = 6 studies). (DOCX 406 kb)

Additional file 7: Grade Table. (DOCX 19 kb)

Additional file 8: Advantages and Disadvantages of Laboratory based EIAs vs RDTs. (DOCX 14 kb)

## Abbreviations

CI: Confidence interval
DAAs: Direct acting antivirals
EIA: Enzyme immunoassay
FN: False negative
FP: False positive
HCV Ab: HCV antibody
HCV: Hepatitis C Virus
NAT: Nucleic acid testing
NPV: Negative predictive value
POC: Point-of-care
PPV: Positive predictive value,
RCT: Randomised control trial
RDTs: Rapid diagnostic tests
SE: Sensitivity
SP: Specificity
TN: True negative
TP: True Positive
WHO: World Health Organization
