# Supplementary figures and images for: Diagnostic accuracy of tests to detect Hepatitis C antibody: a meta-analysis and review of the literature
Source: BMC Infect Dis. 2017 Nov 1;17(Suppl 1):695. doi: 10.1186/s12879-017-2773-2 (PMC5688422; doi:10.1186/s12879-017-2773-2)

**Additional File 1. Pooled test accuracy of HCV Ab RDTs compared to an EIA reference (5 studies).**


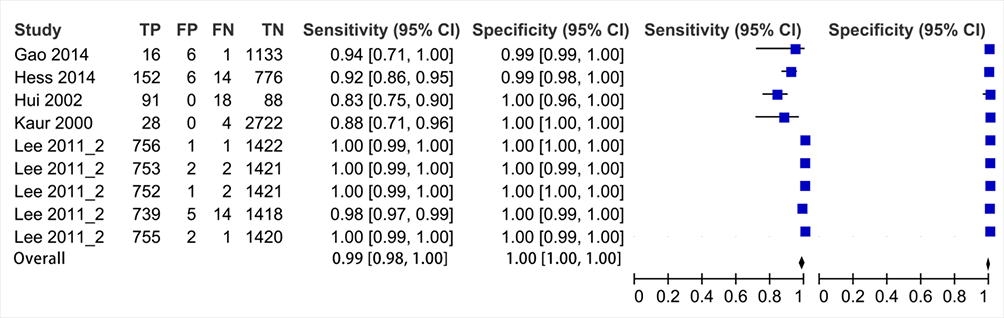

Supplement: Supplementary file 1 — Pooled test accuracy of HCV Ab RDTs compared to an EIA reference (5 studies). (DOCX 360 kb) [file 12879_2017_2773_MOESM1_ESM.docx]

**Additional File 5. Pooled test accuracy for HCV Ab OraQuick kits (n = 8 studies).**

**
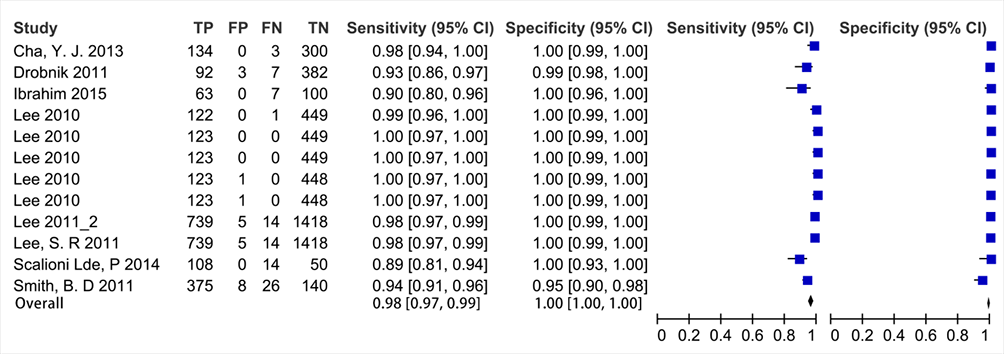
**

Supplement: Supplementary file 5 — Pooled test accuracy for HCV Ab OraQuick kits (n = 8 studies). (DOCX 414 kb) [file 12879_2017_2773_MOESM5_ESM.docx]

**Additional File 6. Pooled test accuracy for other brands of oral HCV Ab test kits (n = 6 studies).**

**
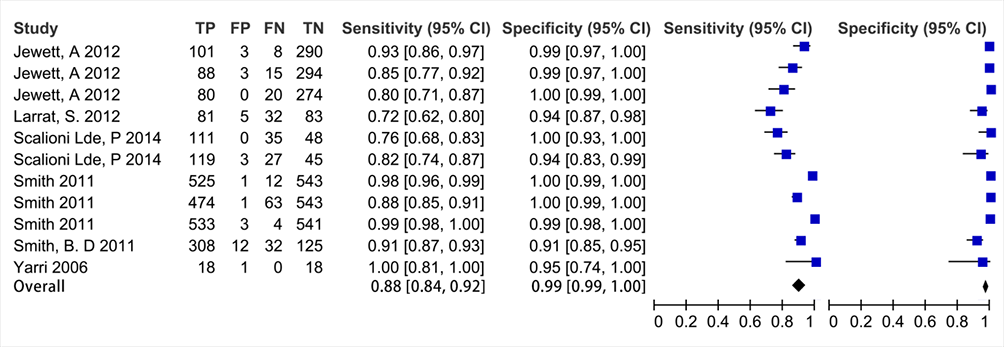
**

Supplement: Supplementary file 6 — Pooled test accuracy for other brands of oral HCV Ab test kits (n = 6 studies). (DOCX 406 kb) [file 12879_2017_2773_MOESM6_ESM.docx]
