# Supplementary material for: Diagnostic accuracy of tests to detect Hepatitis C antibody: a meta-analysis and review of the literature
Source: BMC Infect Dis. 2017 Nov 1;17(Suppl 1):695. doi: 10.1186/s12879-017-2773-2 (PMC5688422; doi:10.1186/s12879-017-2773-2)
Supplement: Supplementary file 2 — Pooled test accuracy of HCV Ab RDTs compared to a NAT or immunoblot reference (n = 13 studies). (DOCX 621 kb) [file 12879_2017_2773_MOESM2_ESM.docx]

**Additional File 2. Pooled test accuracy of HCV Ab RDTs compared to a NAT or immunoblot reference (n = 13 studies).**

**
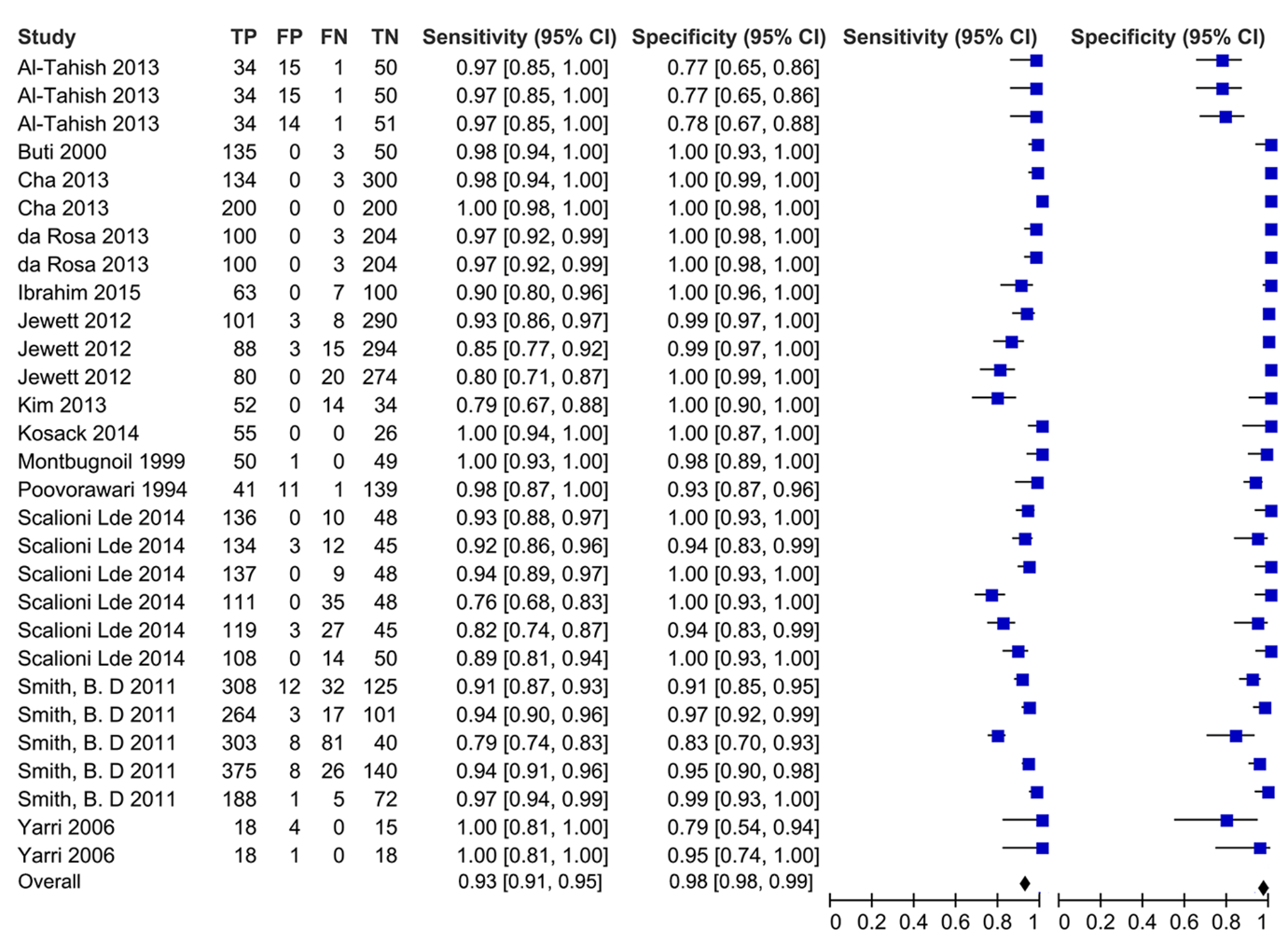
**
