# Supplementary material for: Diagnostic accuracy of tests to detect Hepatitis C antibody: a meta-analysis and review of the literature
Source: BMC Infect Dis. 2017 Nov 1;17(Suppl 1):695. doi: 10.1186/s12879-017-2773-2 (PMC5688422; doi:10.1186/s12879-017-2773-2)
Supplement: Supplementary file 3 — Pooled test accuracy of HCV Ab RDTs compared to EIA, NAT or immunoblot reference standards (n = 14 studies). (DOCX 1170 kb) [file 12879_2017_2773_MOESM3_ESM.docx]

**Additional File 3. Pooled test accuracy of HCV Ab RDTs compared to EIA, NAT or immunoblot reference standards (n = 14 studies).**

**
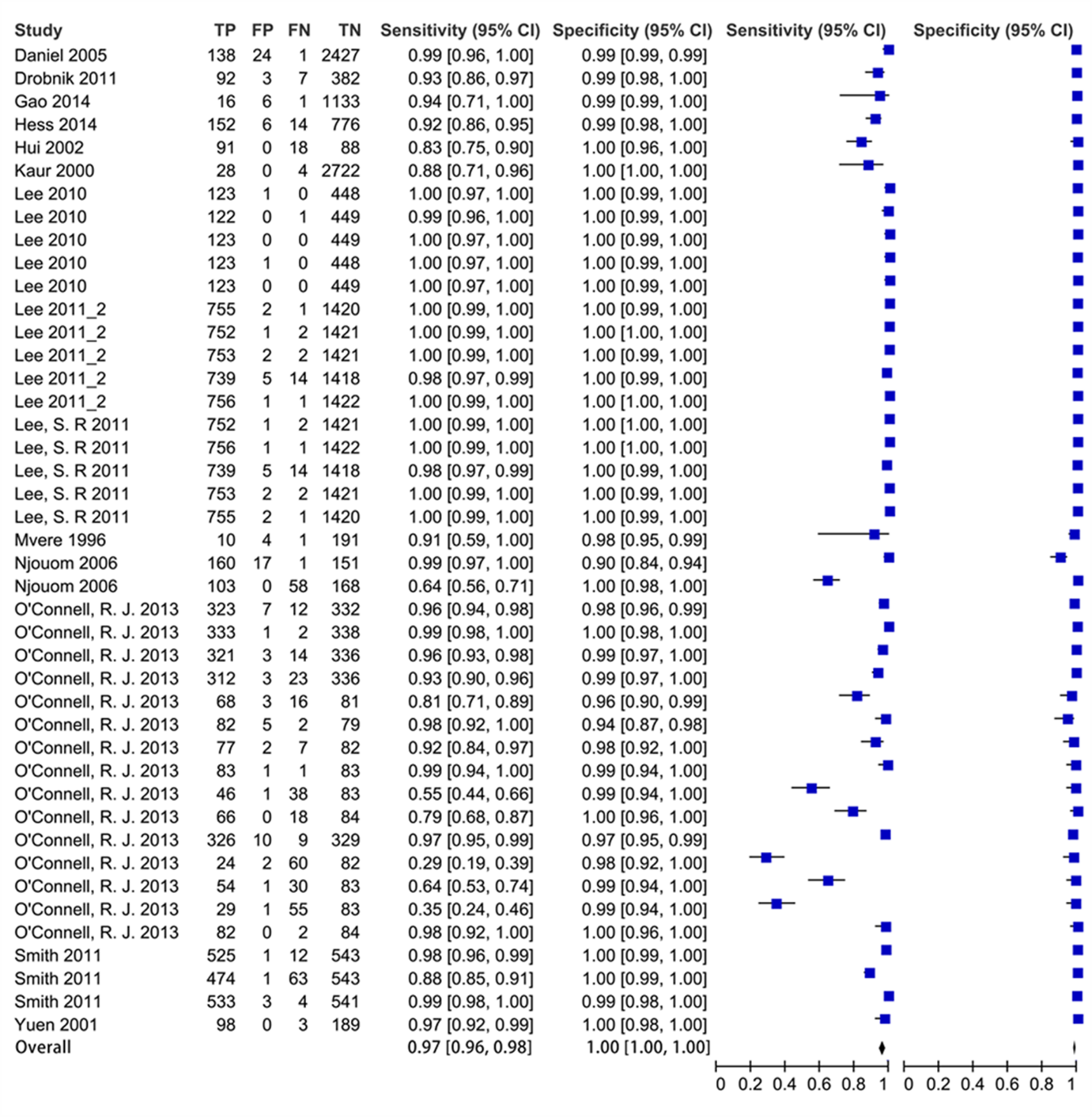
**
