# Supplementary material for: Diagnostic accuracy of tests to detect Hepatitis C antibody: a meta-analysis and review of the literature
Source: BMC Infect Dis. 2017 Nov 1;17(Suppl 1):695. doi: 10.1186/s12879-017-2773-2 (PMC5688422; doi:10.1186/s12879-017-2773-2)
Supplement: Supplementary file 7 — Grade Table. (DOCX 19 kb) [file 12879_2017_2773_MOESM7_ESM.docx]

**Additional file 7 Grade Table**

|  | **RDT versus EIA** | **Oral RDT versus blood** |
| --- | --- | --- |
| **Unit of analysis** | Hospital patients, blood donors, injection drug users and other high risk populations | General population, Hospital patients, blood donors, injection drug users and other high risk population |
| **Sample Type** | Oral fluid serum or plasma | |
| **Studies, *n*** | 5 | 12 |
| **Risk of bias** | Moderate | |
| **Consistency** | Se: Inconsistent Sp: Inconsistent | |
| **Directness/ Precision** | Indirect/ Precise | |
| **# of samples** | 15943 | 14547 |
| **Strength of evidence** | Se: Moderate Sp: Moderate | |
| **Sensitivity (95% CI)** | 99%(98%-100%) | 94%(93%-96%) |
| **Specificity (95%CI)** | 100% (100%-100%) | |
| **Pretest probability (%)** | 5% | |
| **Positive LR (95% CI)** | 618.5(350.6-2493.2) | 314.5 (202.0-684.1) |
| **PPV** | 97% | 94% |
| **Negative LR (95% CI)** | 1% (0.2-2%) | 6% (4%-7%) |
| **NPV** | 1 | |
