# Supplementary material for: Diagnostic accuracy of tests to detect Hepatitis C antibody: a meta-analysis and review of the literature
Source: BMC Infect Dis. 2017 Nov 1;17(Suppl 1):695. doi: 10.1186/s12879-017-2773-2 (PMC5688422; doi:10.1186/s12879-017-2773-2)
Supplement: Supplementary file 8 — Advantages and Disadvantages of Laboratory based EIAs vs RDTs. (DOCX 14 kb) [file 12879_2017_2773_MOESM8_ESM.docx]

**Additional File 8. Advantages and Disadvantages of Laboratory based EIAs vs RDTs.**

|  | **Laboratory-based EIA** | **RDTs** |
| --- | --- | --- |
| **Advantages** | - Accurate - High throughput - Objective, automated reading of results - Within-assay quality control | - Accessible to lowest level of the health care system (including outreach) - Can be used with non-invasive specimens, and facilitate self-testing - Rapid result to enable treatment initiation at the same clinic visit - Can be stored at ambient temperature |
| **Disadvantages** | - Requires laboratory facility, equipment and highly trained staff - Reagents need refrigeration - Need venepuncture to obtain sera - Time to result = 3-4 hours so patients need to return for results | - Lower accuracy than EIAs - Subjective reading and interpretation of results - No built-in quality control - Higher cost/test |
